# acACS: Improving the Prediction Accuracy of Protein Subcellular Locations and Protein Classification by Incorporating the Average Chemical Shifts Composition

**DOI:** 10.1155/2014/864135

**Published:** 2014-07-02

**Authors:** Guo-Liang Fan, Yan-Ling Liu, Yong-Chun Zuo, Han-Xue Mei, Yi Rang, Bao-Yan Hou, Yan Zhao

**Affiliations:** ^1^Department of Physics, School of Physical Science and Technology, Inner Mongolia University, Hohhot 010021, China; ^2^The Key Laboratory of Mammalian Reproductive Biology and Biotechnology of the Ministry of Education, College of Life Sciences, Inner Mongolia University, Hohhot 010021, China

## Abstract

The chemical shift is sensitive to changes in the local environments and can report the structural changes. The structure information of a protein can be represented by the average chemical shifts (ACS) composition, which has been broadly applied for enhancing the prediction accuracy in protein subcellular locations and protein classification. However, different kinds of ACS composition can solve different problems. We established an online web server named acACS, which can convert secondary structure into average chemical shift and then compose the vector for representing a protein by using the algorithm of auto covariance. Our solution is easy to use and can meet the needs of users.

## 1. Introduction

Knowledge of subcellular localization information of a protein may help to unravel its normal cellular function [1]. The proteins within the different compartments have different biological activity and functions; in turn, knowing the subcellular localization of a given protein helps in elucidating its functional role.

Recently, many computational approaches for subcellular localization predictions have been developed and plenty of methods for improving the accuracy of the prediction were applied. From two aspects the predictor can be described. One is the predicting algorithms, like support vector machine (SVM) [2–11], neural network [12], increment of diversity (ID) [13], random forest (RF) [14], K-nearest neighbor (K-NN) [15, 16], generating algorithm [17], and so on, or the combination of them [16, 18]. The other is the information source, such as widely used sequence-based information source, which are amino acid composition (AAC) and sorting signals [19–21], and textual descriptions of proteins [22, 23], which are protein physiochemical property [24], gene ontology (GO) [25], and so on. Actually, the structure information of a protein is very important, especially when it is used for representing the subcellular locations of a protein. However, the structure information of a protein cannot be easily described, and few methods using the structure information can be learned to our knowledge.

However, in NMR spectroscopy, as an important parameter, chemical shift, which is sensitive to changes in the local environments, can report the structural changes. Sibley et al. [26], Mielke and Krishnan [27], Spera and Bax [28], and Zhao et al. [29] have found that the ACS of a protein has intrinsic correlation with the protein's secondary structure and the function of this protein is determined by its structure. According to this point of view, there must be some relationship among the averaged chemical shift, protein structure, and functions [30, 31]. Wishart has developed a web server, namely, CS23D, for rapidly generating accurate 3D protein structures using only assigned NMR chemical shifts [32]. More than 100 proteins from BMRB [33] were tested and found that the resulting structures generally exhibit good geometry and chemical shift agreement [32]. Also, there are some algorithms, which can predict the chemical shift from protein sequences and conformation [34–37]; few works have been done to determine a protein's functions by the chemical shifts [38, 39]. Therefore, how to use the chemical shift is still important and urgent.

In this paper, a benchmark data set of chemical shift was constructed, which consists of 1,552 proteins derived from BMRB website [33] and then extracted chemical shift values of _  _^15^N, _  _^13^C_*α*_, _  _^1^H_*α*_, and _  _^1^H_*N*_ for 20 amino acid residues. Then four types of average chemical shift for 20 amino acid residues were calculated and the autocovariance algorithm was used to convert the average chemical shift into the vector to describe the protein sample. The algorithm acACS (autocovariance of averaged chemical shifts) has been used to enhance the prediction accuracy in protein subcellular locations. The proposed acACS descriptor can be considered as a mode of generalized pseudoamino acid composition, which was summarized in [40]. Recently, the generalized pseudoamino acid composition methods have been systematically implemented by two powerful software, PseAAC-Builder [41] and PseAAC-General [42]. For the readers' convenience in using the current method, the acACS descriptor may be integrated into this software in future works. The details of how to deal with this calculation and how to use this method is shown as follows.

## 2. Material and Methods 

### 2.1. Data Sets

When an electron moves around a proton, it will produce some magnetic field, which could affect proton's external electron field. Thus, the absorption frequencies of proton in different chemical environments would shift relatively to the absorption frequencies under standard magnetic fields. Chemical shift is the relative resonance frequencies shift of protons between different chemical environment and standard, which can be measured by NMR spectroscopy. Due to its sensitivity to local environments, such as the backbone dihedral angles and the secondary structure types [26, 27, 29], chemical shift can be an indicator for the changes of local conformations.

In order to find out the correlation between chemical shift and the secondary structure of a protein, we construct a high-quality working data set, which started from the following steps: (1) the proteins star file with NMR spectroscopy data were downloaded from BMRB [33]; (2) the proteins less than 50 residues or not matched to PDB [43] entries were discarded; (3) the proteins with sequence identity higher than 40% were excluded by CD-HIT [44]. Finally, the benchmark data set has 1,552 proteins. The data set was available at our website. The data set contained 1,552 proteins sequences and BMRB star file, which was the original chemical shifts data file for all kinds of backbone atoms of each protein. We analyzed the averaged chemical shifts for every kind of amino acids type and secondary structure in order to find out the rules among averaged chemical shifts with every kind of amino acids type and secondary structure types and then used the autocovariance algorithm to calculate the feature vectors of the protein sequences from the statistic results. The feature vectors representing the protein sequences can be used in problems of subcellular location prediction or other protein classifications. Researchers may also develop better algorithms for protein representation using the data set.

### 2.2. Averaged Chemical Shift (ACS)

In order to find the rule between the chemical shifts and structure information, the statistic about averaged chemical shift related to secondary structure and amino acids type was carried out.

Firstly, four types chemical shift values *ω* of _  _^15^N, _  _^13^C_*α*_, _  _^1^H_*α*_, and _  _^1^H_*N*_ from every amino acid residue were extracted from the BMRB star file for further calculation. In the BMRB star file, the amino acid residues, four kinds of protein backbone atoms of each amino acid residue, and matched PDB file were given. For example, the “bmr447.str” was extracted into four files: N_447.txt, Ca_447.txt, Ha_447.txt, and Hn_447.txt, which correspond to _  _^15^N, _  _^13^C_*α*_, _  _^1^H_*α*_, and _  _^1^H_*N*_ protein backbone atoms.

Secondly, the secondary structure information was extracted from PDB file which matched to BMRB star file. The secondary structure types of each amino acid residue are denoted by H, E, and C. Then the averaged chemical shifts for all the residues were calculated.

For protein backbone atoms “*i*” of amino acid type “*j*” with secondary structure type “*k*,” the averaged chemical shift (ACS) is defined as

(1) $$\begin{array}{c} C_{k}^{i}\left(j\right)=\frac{1}{N}\underset{N}{\sum }\omega_{k}^{i}\left(j\right). \end{array}$$

Here *i* = ^15^N, _  _^13^C_*α*_, _  _^1^H_*α*_, or _  _^1^H_*N*_, *j* is one kind of 20 amino acids and *k* stands for the secondary structure types (H, E, or C) from DSSP [45] (H = helix, E = strand, and C = the rest). *ω*_*k*_^*i*^(*j*) is the chemical shift value extracted from the BMRB star file and *N* is the counts of *ω*_*k*_^*i*^(*j*) items.

**Figure 1:**
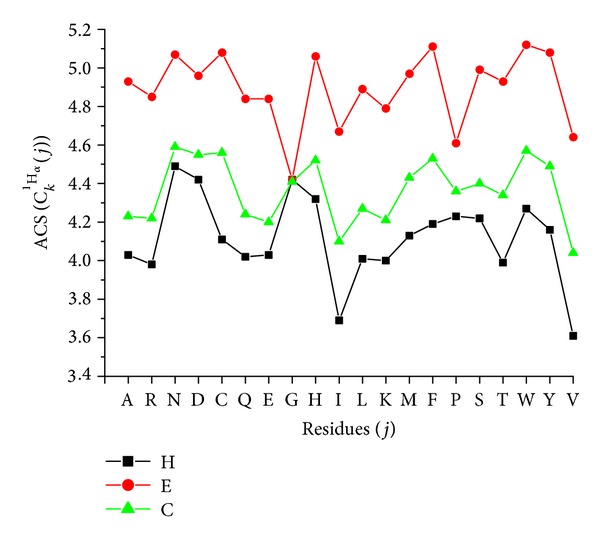
The average chemical shifts (ACS) of _  _^1^H_*α*_ for each of 20 native amino acid residues (*j*) with three types of secondary structure (*k*).

**Figure 2:**
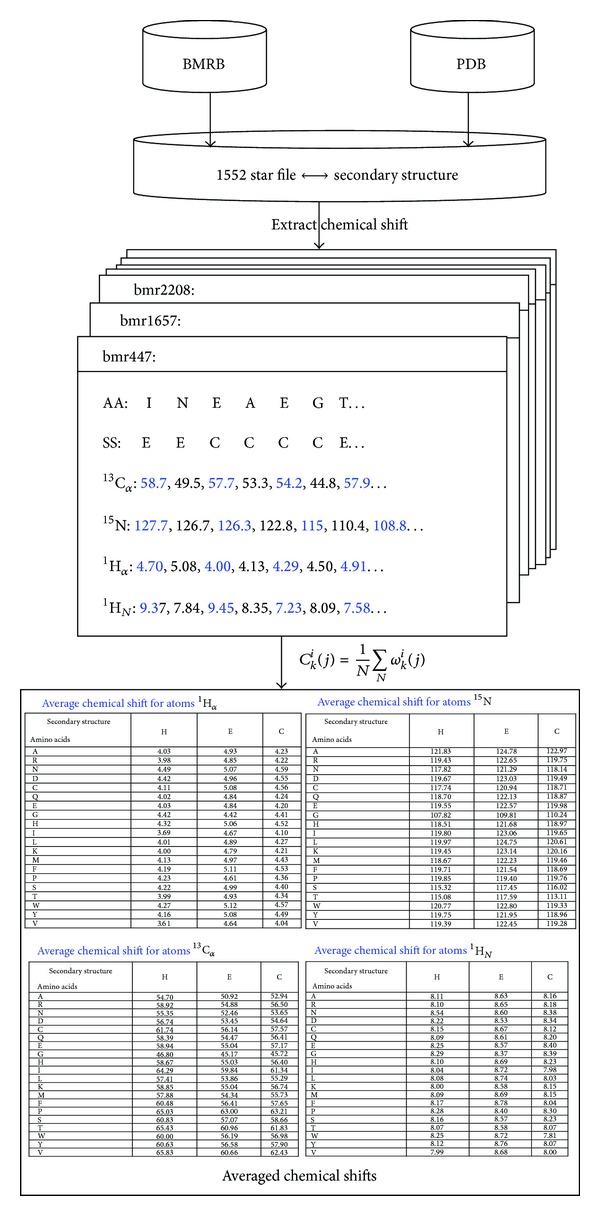
The flowchart of calculating the ACS. The AA denotes the amino acids and the SS denotes the secondary structure.

By calculating the residues' ACS with (1) for 1552 proteins, we found that the ACS regularly varies with the secondary structure types and residues. The statistic results of averaged chemical shifts were listed in four tables, which can be accessed from our website. Take the _  _^1^H_*α*_ as an example, the ACS of _  _^1^H_*α*_ for each of 20 native amino acid residues with three types of secondary structure is shown in Figure 1. According to Figure 1, it can be concluded that we can use the ACS to represent the protein's secondary structure. In order to illustrate the algorithm, the flowchart of ACS is given in Figure 2.

### 2.3. Algorithm of Autocovariance of Average Chemical Shift (acACS)

In order to obtain the correlation information between amino acids of a protein, the autocovariance of ACS was calculated. For a protein *P*,

(2) $$\begin{array}{c} P=\left[j_{1},j_{2}\cdots j_{l}\cdots j_{L}\right]. \end{array}$$

Here, *L* is the sequence length and *j*_*l*_ is the amino acid in position *l*.

The secondary structure of protein *P* was predicted from Porter [46, 47] and then

(3) $$\begin{array}{c} P=\left[k_{1},k_{2}\cdots k_{l}\cdots k_{L}\right]. \end{array}$$

Here *k* is the secondary structure types.

Then, the amino acid *j*_*l*_ in protein *P* was replaced by its ACS “*C*_*k*_*l*__^*i*^(*j*_*l*_)” according to its secondary structure type *k*_*l*_. When *C*_*k*_*l*__^*i*^(*j*_*l*_) was redefined as *S*_*l*_^*i*^, *P* can be expressed as

(4) P=[S1i,S2i⋯Sli⋯SLi] (i=N15,Cα  13,Hα  1,HN  1).

Then, the autocovariance algorithm was used to calculate the correlation between amino acid *l* and *l* + *λ* by the following equation:

(5) θi(λ)=1L−λ∑l=1L−λ[Sli−S(l+λ)i]2,(i=N15,Cα  13,Hα  1,HN  1,  0<λ<L).

After the above calculation, the protein *P* can be expressed as follows:

(6) PacACS=[θi(0),θi(1),θi(2),θi(3),…,θi(λ);…](i=N15,Cα  13,Hα  1,HN  1,  0<λ<L).

Here, *θ* ^*i*^(*λ*) is the correlation factor of average chemical shift *S*_*l*_^*i*^ with average chemical shift *S*_*l*+*λ*_^*i*^. In particular, when *λ* = 0, with (5), *θ* ^*i*^(0) = 0. In order to take use of ACS, the *θ* ^*i*^(0) was replaced by the average chemical shift *S*_*l*_^*i*^. The factor *λ* is a nonnegative integer and reflects the rank of correlation [40]. Based on different problems, in order to get a best result, a certain right number for factor *λ* should be given and so does *i*.

**Figure 3:**
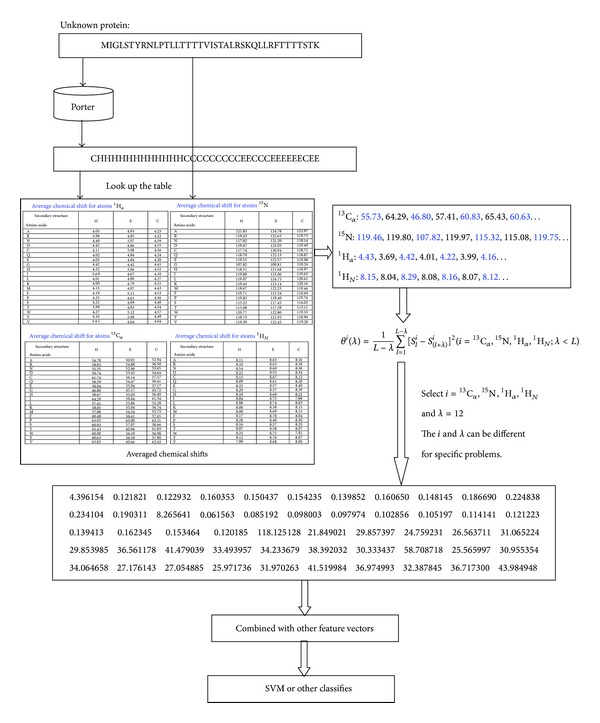
The flow diagram of the processing of the acACS.

In order to give a pictorial representation of chemical shifting technique, a flow diagram is given in Figure 3, which shows how the acACS works.

## 3. Results and Discussion

By using the acACS algorithm, we successfully represented the protein samples and accurately predicted submitochondria locations. We used the model to test the SML3-983 data set that was along with the SubMito-PSPCP [48]. The data set has 983 proteins sequences which were divided into three locations. Among the data set, there are 661 sequences from inner membrane, 177 sequences from matrix, and 145 sequences from outer membrane. We selected acACS combined with AAC, DC, PSSM, and GO and reduced physicochemical properties (Hn) as feature vectors for representing the proteins and then trained the model. Then 90.74% accuracy was obtained for SML3-983 data set with Jackknife cross-validation, which was 1.63% higher than SubMito-PSPCP. In order to compare the performance of acACS, the feature vector was recombined with AAC, DC, PSSM, GO, and Hn, without acACS. Then we trained the model and obtained the predicting accuracy of 89.52%, which was dropped about 1.2%.

The acACS algorithm has also been checked in our previous works [49–52]. In subcellular location prediction, we compared the results with and without the acACS in the submitochondria locations and mycobacterial proteins subcellular locations and got the better result which was listed in Tables 1 and 2. Actually, the acACS as a feature vector for representing the protein samples can also be used for other kinds of proteins prediction problem. In acidic and alkaline enzymes prediction and bioluminescent and nonbioluminescent proteins discrimination, we also improved the predicting accuracy by about 1.3%, which was listed in Table 3.

The protein functions, including its subcellular locations, are largely determined by its structure. Developing a novel method for improving the performance of predicting protein subcellular locations is urgent. However, the feature vectors in the methods were almost sequence-based in the past. Therefore, almost state-of-the-art methods tried to incorporate some other sequence-based information as its complement. Our method provides structure-based information and can be perfect complement to the sequence-based methods and can be used for other kinds of protein related problems. Actually, these methods can work side by side to help each other in a practical study.

For the chemical shift, it incorporates the structure information in the first place, so it can represent the protein sample better. What is the better ways to use of the chemical shift is still a hot topic for biologist and chemist. In this work, we used the autocovariance algorithm to process the averaged chemical shift and got the better results, but there are certainly some improvements that could be made for acACS. Actually other algorithms can be adopted to try to find the better method for representing the protein samples in the future. At present stage, it is not convenient for the user, for both the secondary structure information and the protein sequence that are used, to calculate the chemical shift. In the future, the secondary structure information will not be necessary, and it will be integrated into the algorithm.

## 4. Conclusions

In this work, the raw chemical shifts data set and averaged chemical shift data set were constructed. Then, the averaged chemical shift was calculated and the algorithm of acACS was presented. In order to check the performance of the acACS we got, proteins submitochondria locations, mycobacterial proteins subcellular locations, bioluminescent proteins discrimination, and acidic and alkaline enzymes classification were predicted. Based on the results we obtain, it can be concluded that the acACS can improve the accuracy of prediction at least 1%-2%, the performance of which is correlated with the correlation factor *λ* and the backbone atoms *i*. Some recent studies showed that the profile-based features [54–56], pseudoamino acid composition (PseAAC) [57], and features based on physicochemical proprieties of amino acids [58] were able to improve the performance of many computational predictors for protein remote homology detection, protein binding site identification, and so forth. Therefore, these features should be studied for protein subcellular location prediction in the future studies.

We have developed a web server acACS, which could automatically produce the vectors of proteins, when a custom submitted the protein sequences along with the secondary structure in batch mode. The data set can be a very useful addition to biomolecular NMR spectroscopists. The acACS will be of benefit to the proteomic research. The current work will become an important progress in the prediction of the protein subcellular locations and promote the study in the related areas.

## 5. Web Server and User Guide 

To enhance the value of its practical applications, a web server for the acACS generator was established. Moreover, for the convenience of the user, here a step-to-step guide is provided for how to use the web server to get the desired results.

**Figure 4:**
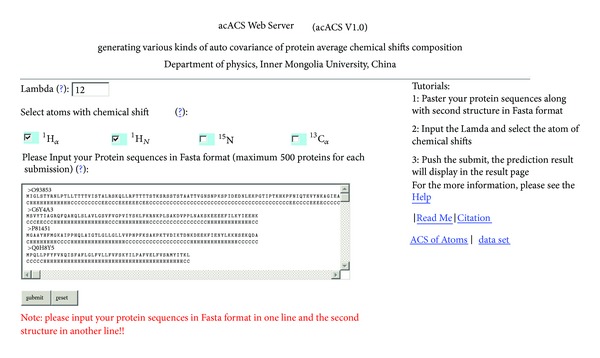
A screenshot for the top page of the web server acACS at http://wlxy.imu.edu.cn/college/biostation/fuwu/acACS/index.asp.

*Step 1.* Open the web server at http://wlxy.imu.edu.cn/college/biostation/fuwu/acACS/index.asp and you will see the top page of the acACS on your computer screen, as shown in Figure 4. Click on the Read Me button to see a brief introduction about the acACS.

*Step 2*. Either type or copy/paste the query protein sequences into the input box at the center of Figure 4, and then copy/paste the secondary structure of the protein sequence in the next line. The input sequence should be in “ONE LINE” format. For the examples of sequences in ONE LINE format, click the “?” button above the input box.

*Step 3*. Input the Lambda value in the input box right of the Lambda label.

*Step 4*. Check atoms with chemical shift.

*Step 5*. Click on the Submit button to see the result page. For example, if you use the default example sequences, Lambda and atoms in the window, after clicking the Submit button, you will see the following message shown on the screen of your computer: “The lamda you have chosen is 12”; “The Atom of chemical shift you have chosen are _  _^1^H_*α*_,_  _^1^H_*N*_”; “The acACSs of the proteins you submitted are......”. Then the acACS of _  _^1^H_*α*_ atom was given and the acACS of _  _^1^H_*N*_ atom followed for the first protein, then the acACS of second protein, the third, and so forth.

*Step 6*. Click the ACS of atoms and data set button to download the benchmark dataset used to calculate the ACS.

*Step 7*. Click the Citation button to find the relevant papers that document the detailed development and algorithm of acACS.

## Figures and Tables

**Table 1:** The comparison of the results with and without the acACS for predicting submitochondria locations and three membrane protein types with comparison to that without acACS.

	With acACS	Without acACS
Submitochondria locations	93.57%	91.46%
Three membrane protein types	97.79%	96.10%
Data set of Du [24]	94.95%	93.43%

**Table 2:** The comparison of the results with and without the acACS for predicting mycobacterial subcellular localizations and three membrane protein types.

	With acACS	Without acACS
Mycobacterial subcellular localizations	87.77%	86.19%
Three membrane protein types	85.03%	83.71%
Data set of Rashid [53]	98.12%	96.85%

**Table 3:** The comparison of the results with and without the acACS for other kinds of proteins prediction.

	With acACS	Without acACS
Acidic and alkaline enzymes	94.01%	92.52%
Bioluminescent and nonbioluminescent proteins	82.16%	80.90%
